# (*Z*)-1-(3-Nitro­phen­yl)-2-(4-nitro­phen­yl)ethene

**DOI:** 10.1107/S1600536808021077

**Published:** 2008-07-12

**Authors:** Chenzhong Cao, Liqiu Liu

**Affiliations:** a School of Chemistry and Chemical Engineering, Hunan University of Science and Technology, Xiangtan 411201, People’s Republic of China

## Abstract

In the mol­ecule of the title compound, C_14_H_10_N_2_O_4_, the dihedral angle formed by the benzene rings is 53.66 (5)°. In the crystal structure, mol­ecules are linked into chains parallel to the [0
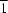
1] direction by inter­molecular C—H⋯O hydrogen-bonding inter­actions.

## Related literature

For related literature, see: Boonlaksiri *et al.* (2000 ▶); Papper & Likhtenshtein (2001 ▶); Soto Bustmante *et al.* (1995 ▶). For the crystal structure of a related isomer, see: Chen & Cao (2007 ▶).
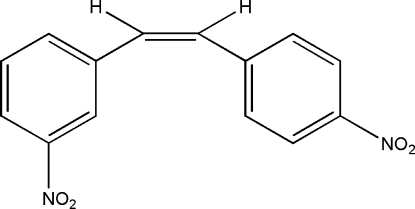


## Experimental

### 

#### Crystal data

C_14_H_10_N_2_O_4_
*M* *_r_* = 270.24
Triclinic, 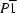

*a* = 7.2995 (13) Å
*b* = 8.0561 (11) Å
*c* = 11.831 (2) Å
α = 78.291 (7)°
β = 85.102 (7)°
γ = 67.536 (7)°
*V* = 629.53 (18) Å^3^
*Z* = 2
Mo *K*α radiation
μ = 0.11 mm^−1^
*T* = 296 (2) K
0.50 × 0.24 × 0.19 mm

#### Data collection

Bruker APEX CCD diffractometer
Absorption correction: multi-scan (*SADABS*; Sheldrick, 2002 ▶) *T*_min_ = 0.946, *T*_max_ = 0.981
4608 measured reflections
2902 independent reflections
2009 reflections with *I* > 2σ(*I*)
*R*_int_ = 0.021

#### Refinement

*R*[*F* ^2^ > 2σ(*F* ^2^)] = 0.046
*wR*(*F* ^2^) = 0.138
*S* = 1.03
2902 reflections
182 parameters
H-atom parameters constrained
Δρ_max_ = 0.24 e Å^−3^
Δρ_min_ = −0.19 e Å^−3^

### 

Data collection: *SMART* (Bruker, 2003 ▶); cell refinement: *SAINT* (Bruker, 2003 ▶); data reduction: *SAINT*; program(s) used to solve structure: *SHELXS97* (Sheldrick, 2008 ▶); program(s) used to refine structure: *SHELXL97* (Sheldrick, 2008 ▶); molecular graphics: *SHELXTL* (Sheldrick, 2008 ▶); software used to prepare material for publication: *SHELXTL*.

## Supplementary Material

Crystal structure: contains datablocks I, global. DOI: 10.1107/S1600536808021077/rz2231sup1.cif

Structure factors: contains datablocks I. DOI: 10.1107/S1600536808021077/rz2231Isup2.hkl

Additional supplementary materials:  crystallographic information; 3D view; checkCIF report

## Figures and Tables

**Table 1:** Hydrogen-bond geometry (Å, °)

*D*—H⋯*A*	*D*—H	H⋯*A*	*D*⋯*A*	*D*—H⋯*A*
C13—H13*A*⋯O1^i^	0.93	2.56	3.388 (2)	149

Symmetry code: (i) 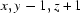.

**Fig. 1.**
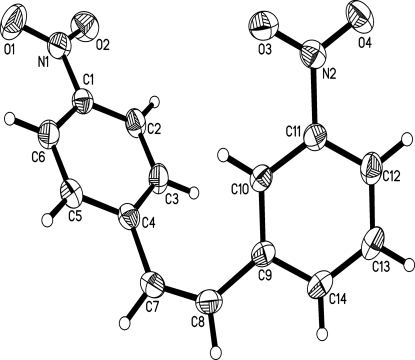
The molecular structure of the title compound with the atom-numbering scheme and displacement ellipsoids drawn at the 30% probability level.

**Fig. 2.**
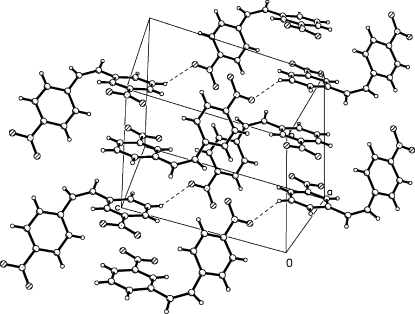
Packing diagram of the title compound showing intermolecular hydrogen bonds (dashed lines) forming chains parallel to the [0 -1 1] direction.

## supplementary crystallographic information

### Comment

Recently, stilbene derivatives have attracted considerable attention from chemists and biologists because of their non-linear optical properties (Soto Bustmante *et al.*, 1995; Papper & Likhtenshtein, 2001) and biological activities (Boonlaksiri *et al.*, 2000). The crystal structure of the related isomer (Z)-1,2-bis(4-nitrophenyl)ethene has been previously reported by our group (Chen & Cao, 2007). We report here the crystal structure of the title compound (Fig. 1), a *cis*-stilbene derivative.

In the title compound, the C4—C7—C8 and C9—C8—C7 bond angles are 130.11 (16) and 129.92 (15)°, respectively. They are larger than the idealized value of 120° expected for *sp*^2 ^hybrid orbitals due to the comparatively strong stereo hindrance between the two aryl groups. The dihedral angle between the two benzene rings is 53.66 (5)°. The nitro groups at C1 and C11 are slightly twisted out of the plane of the attached benzene rings forming dihedral angles of 7.92 (14) and 9.22 (10)°, respectively. In the crystal structure (Fig. 2), there is non-classical intermolecular C—H···O hydrogen bond (Table 1) linking molecules into chains running parallel to the [0 -1 1] direction.

### Experimental

The title compound was synthesized by the Wittig reaction. Triphenyl(*p*-nitrobenzyl)phosphonium chloride (0.01 mol), which was obtained by reacting 4-nitrobenzyl chlorine with triphenyl phosphine, and 3-nitrobenzaldehyde (0.01 mol) were dissolved in CH_2_Cl_2_ (15 ml), then a 50% NaOH solution (4 ml) was titrated into the mixture. The mixture was refluxed for 40 min at 45–50 °C. After cooling to room temperature, water (15 ml) was added and the mixture was extracted with ether (20 ml). The organic layer was washed with water and dried with anhydrous sodium sulfate, then it was filtered and concentrated. The resulting yellow solution was collected and purified by column chromatography on silica gel using petroleum ether and chloroform (10:1 v/v) as eluent (yield: 8.6%). Crystals of the title compound suitable for X-ray analysis were grown by slow evaporation of an ethanol solution. ^1^HNMR (CDCl_3_)(400 MHz; TMS p.p.m.), δ(p.p.m.): 6.83–6.90 (m, 2H, –C═C–), 7.31–7.54 (m, 4H, Ar), 8.13–8.17 (m, 4H, Ar).

### Refinement

The hydrogen atoms were generated geometrically and refined using a riding model, with C—H = 0.93 Å and *U*_iso_(H) = 1.2 *U*_eq_(C).

### Crystal data

**Table:**

C_14_H_10_N_2_O_4_	*Z* = 2
*M**_r_* = 270.24	*F*_000_ = 280
Triclinic, *P*1	*D*_x_ = 1.426 Mg m^−^^3^
Hall symbol: -P 1	Mo *K*α radiation λ = 0.71073 Å
*a* = 7.2995 (13) Å	Cell parameters from 1175 reflections
*b* = 8.0561 (11) Å	θ = 3.5–27.2º
*c* = 11.831 (2) Å	µ = 0.11 mm^−^^1^
α = 78.291 (7)º	*T* = 296 (2) K
β = 85.102 (7)º	Block, yellow
γ = 67.536 (7)º	0.50 × 0.24 × 0.19 mm
*V* = 629.53 (18) Å^3^	

### Data collection

**Table:**

Bruker SMART APEXII CCD diffractometer	2902 independent reflections
Radiation source: fine-focus sealed tube	2009 reflections with *I* > 2σ(*I*)
Monochromator: graphite	*R*_int_ = 0.021
*T* = 296(2) K	θ_max_ = 27.9º
ω scans	θ_min_ = 1.8º
Absorption correction: multi-scan(SADABS; Sheldrick, 2002)	*h* = −9→9
*T*_min_ = 0.946, *T*_max_ = 0.981	*k* = −10→9
4608 measured reflections	*l* = −15→15

### Refinement

**Table:**

Refinement on *F*^2^	Hydrogen site location: inferred from neighbouring sites
Least-squares matrix: full	H-atom parameters constrained
*R*[*F*^2^ > 2σ(*F*^2^)] = 0.047	*w* = 1/[σ^2^(*F*_o_^2^) + (0.066*P*)^2^ + 0.0698*P*] where *P* = (*F*_o_^2^ + 2*F*_c_^2^)/3
*wR*(*F*^2^) = 0.138	(Δ/σ)_max_ < 0.001
*S* = 1.03	Δρ_max_ = 0.24 e Å^−^^3^
2902 reflections	Δρ_min_ = −0.19 e Å^−^^3^
182 parameters	Extinction correction: SHELXL97 (Sheldrick, 2008), Fc^*^=kFc[1+0.001xFc^2^λ^3^/sin(2θ)]^-1/4^
Primary atom site location: structure-invariant direct methods	Extinction coefficient: 0.036 (7)
Secondary atom site location: difference Fourier map	

### Special details

**Table:**

Geometry. All e.s.d.'s (except the e.s.d. in the dihedral angle between two l.s. planes) are estimated using the full covariance matrix. The cell e.s.d.'s are taken into account individually in the estimation of e.s.d.'s in distances, angles and torsion angles; correlations between e.s.d.'s in cell parameters are only used when they are defined by crystal symmetry. An approximate (isotropic) treatment of cell e.s.d.'s is used for estimating e.s.d.'s involving l.s. planes.
Refinement. Refinement of *F*^2^ against ALL reflections. The weighted *R*-factor *wR* and goodness of fit *S* are based on *F*^2^, conventional *R*-factors *R* are based on *F*, with *F* set to zero for negative *F*^2^. The threshold expression of *F*^2^ > σ(*F*^2^) is used only for calculating *R*-factors(gt) *etc*. and is not relevant to the choice of reflections for refinement. *R*-factors based on *F*^2^ are statistically about twice as large as those based on *F*, and *R*- factors based on ALL data will be even larger.

### Fractional atomic coordinates and isotropic or equivalent isotropic displacement parameters (Å^2^)

**Table:**

	*x*	*y*	*z*	*U*_iso_*/*U*_eq_	
C11	0.4027 (2)	0.3521 (2)	0.95493 (13)	0.0408 (4)	
C10	0.5246 (2)	0.2712 (2)	0.87050 (13)	0.0407 (4)	
H10A	0.4941	0.3190	0.7930	0.049*	
N2	0.2245 (2)	0.51528 (19)	0.92062 (12)	0.0491 (4)	
O4	0.1303 (2)	0.59945 (18)	0.99507 (11)	0.0625 (4)	
C9	0.6945 (2)	0.1168 (2)	0.90224 (13)	0.0418 (4)	
C14	0.7294 (3)	0.0475 (2)	1.01961 (14)	0.0491 (4)	
H14A	0.8396	−0.0581	1.0424	0.059*	
C4	0.8254 (2)	0.2702 (2)	0.64805 (13)	0.0445 (4)	
C1	0.7410 (2)	0.6180 (2)	0.52136 (13)	0.0437 (4)	
N1	0.7019 (2)	0.8008 (2)	0.45317 (13)	0.0553 (4)	
C12	0.4397 (3)	0.2862 (2)	1.07097 (14)	0.0509 (4)	
H12A	0.3552	0.3448	1.1262	0.061*	
O2	0.7283 (2)	0.91400 (19)	0.49721 (13)	0.0734 (4)	
O3	0.1784 (2)	0.5610 (2)	0.81952 (12)	0.0804 (5)	
C3	0.8289 (3)	0.4109 (2)	0.69887 (14)	0.0518 (4)	
H3A	0.8598	0.3868	0.7767	0.062*	
C6	0.7387 (3)	0.4823 (2)	0.46765 (14)	0.0490 (4)	
H6A	0.7089	0.5071	0.3896	0.059*	
C5	0.7812 (2)	0.3098 (2)	0.53140 (14)	0.0485 (4)	
H5A	0.7803	0.2174	0.4957	0.058*	
C2	0.7876 (3)	0.5846 (2)	0.63620 (14)	0.0509 (4)	
H2A	0.7911	0.6773	0.6707	0.061*	
C13	0.6048 (3)	0.1315 (3)	1.10274 (14)	0.0547 (5)	
H13A	0.6326	0.0834	1.1805	0.066*	
C8	0.8307 (3)	0.0184 (2)	0.81795 (15)	0.0511 (4)	
H8A	0.8927	−0.1070	0.8429	0.061*	
C7	0.8788 (3)	0.0810 (2)	0.71159 (15)	0.0531 (4)	
H7A	0.9588	−0.0085	0.6709	0.064*	
O1	0.6446 (3)	0.8324 (2)	0.35477 (12)	0.0854 (5)	

### Atomic displacement parameters (Å^2^)

**Table:**

	*U*^11^	*U*^22^	*U*^33^	*U*^12^	*U*^13^	*U*^23^
C11	0.0452 (9)	0.0352 (8)	0.0444 (8)	−0.0183 (7)	0.0008 (6)	−0.0066 (6)
C10	0.0452 (8)	0.0378 (8)	0.0383 (7)	−0.0167 (7)	−0.0026 (6)	−0.0025 (6)
N2	0.0481 (8)	0.0430 (8)	0.0556 (8)	−0.0150 (6)	0.0023 (6)	−0.0129 (7)
O4	0.0606 (8)	0.0543 (8)	0.0711 (8)	−0.0164 (6)	0.0168 (6)	−0.0256 (6)
C9	0.0439 (9)	0.0350 (8)	0.0457 (8)	−0.0163 (7)	−0.0029 (6)	−0.0018 (6)
C14	0.0525 (10)	0.0419 (9)	0.0503 (9)	−0.0207 (8)	−0.0091 (7)	0.0066 (7)
C4	0.0398 (8)	0.0452 (9)	0.0452 (8)	−0.0129 (7)	0.0074 (6)	−0.0104 (7)
C1	0.0399 (8)	0.0440 (9)	0.0449 (8)	−0.0156 (7)	0.0068 (6)	−0.0060 (7)
N1	0.0540 (9)	0.0489 (9)	0.0581 (9)	−0.0181 (7)	0.0067 (7)	−0.0047 (7)
C12	0.0675 (11)	0.0484 (10)	0.0420 (8)	−0.0291 (9)	0.0087 (8)	−0.0086 (7)
O2	0.0851 (11)	0.0496 (8)	0.0885 (10)	−0.0295 (7)	0.0047 (8)	−0.0130 (7)
O3	0.0746 (10)	0.0741 (10)	0.0590 (8)	0.0134 (8)	−0.0155 (7)	−0.0145 (7)
C3	0.0642 (11)	0.0569 (11)	0.0373 (8)	−0.0261 (9)	0.0041 (7)	−0.0105 (7)
C6	0.0498 (10)	0.0544 (10)	0.0416 (8)	−0.0181 (8)	−0.0002 (7)	−0.0095 (7)
C5	0.0512 (10)	0.0495 (10)	0.0476 (9)	−0.0185 (8)	0.0046 (7)	−0.0180 (7)
C2	0.0624 (11)	0.0497 (10)	0.0462 (9)	−0.0260 (8)	0.0092 (7)	−0.0157 (7)
C13	0.0726 (12)	0.0543 (10)	0.0384 (8)	−0.0311 (9)	−0.0056 (8)	0.0053 (7)
C8	0.0503 (10)	0.0353 (8)	0.0586 (10)	−0.0082 (7)	−0.0026 (8)	−0.0035 (7)
C7	0.0526 (10)	0.0427 (9)	0.0556 (10)	−0.0085 (8)	0.0078 (8)	−0.0129 (8)
O1	0.1193 (14)	0.0700 (10)	0.0582 (9)	−0.0343 (9)	−0.0165 (8)	0.0116 (7)

### Geometric parameters (Å, °)

**Table:**

C11—C10	1.372 (2)	C1—N1	1.462 (2)
C11—C12	1.377 (2)	N1—O1	1.2150 (19)
C11—N2	1.467 (2)	N1—O2	1.218 (2)
C10—C9	1.392 (2)	C12—C13	1.374 (3)
C10—H10A	0.9300	C12—H12A	0.9300
N2—O3	1.2143 (18)	C3—C2	1.376 (2)
N2—O4	1.2209 (17)	C3—H3A	0.9300
C9—C14	1.393 (2)	C6—C5	1.371 (2)
C9—C8	1.470 (2)	C6—H6A	0.9300
C14—C13	1.378 (3)	C5—H5A	0.9300
C14—H14A	0.9300	C2—H2A	0.9300
C4—C5	1.389 (2)	C13—H13A	0.9300
C4—C3	1.397 (2)	C8—C7	1.328 (2)
C4—C7	1.473 (2)	C8—H8A	0.9300
C1—C2	1.376 (2)	C7—H7A	0.9300
C1—C6	1.378 (2)		
			
C10—C11—C12	122.86 (15)	C13—C12—C11	118.13 (16)
C10—C11—N2	118.83 (13)	C13—C12—H12A	120.9
C12—C11—N2	118.31 (15)	C11—C12—H12A	120.9
C11—C10—C9	119.22 (14)	C2—C3—C4	121.34 (15)
C11—C10—H10A	120.4	C2—C3—H3A	119.3
C9—C10—H10A	120.4	C4—C3—H3A	119.3
O3—N2—O4	123.14 (15)	C5—C6—C1	118.73 (15)
O3—N2—C11	118.50 (14)	C5—C6—H6A	120.6
O4—N2—C11	118.35 (14)	C1—C6—H6A	120.6
C10—C9—C14	117.92 (15)	C6—C5—C4	121.50 (15)
C10—C9—C8	122.99 (14)	C6—C5—H5A	119.2
C14—C9—C8	118.99 (15)	C4—C5—H5A	119.2
C13—C14—C9	121.71 (16)	C3—C2—C1	118.52 (16)
C13—C14—H14A	119.1	C3—C2—H2A	120.7
C9—C14—H14A	119.1	C1—C2—H2A	120.7
C5—C4—C3	117.99 (15)	C12—C13—C14	120.13 (15)
C5—C4—C7	119.46 (15)	C12—C13—H13A	119.9
C3—C4—C7	122.43 (15)	C14—C13—H13A	119.9
C2—C1—C6	121.91 (15)	C7—C8—C9	129.92 (15)
C2—C1—N1	119.00 (15)	C7—C8—H8A	115.0
C6—C1—N1	119.03 (15)	C9—C8—H8A	115.0
O1—N1—O2	123.13 (16)	C8—C7—C4	130.11 (16)
O1—N1—C1	118.13 (16)	C8—C7—H7A	114.9
O2—N1—C1	118.75 (15)	C4—C7—H7A	114.9
			
C12—C11—C10—C9	−0.7 (2)	C7—C4—C3—C2	176.81 (16)
N2—C11—C10—C9	179.75 (13)	C2—C1—C6—C5	1.0 (3)
C10—C11—N2—O3	8.4 (2)	N1—C1—C6—C5	178.16 (14)
C12—C11—N2—O3	−171.23 (16)	C1—C6—C5—C4	0.2 (3)
C10—C11—N2—O4	−171.07 (14)	C3—C4—C5—C6	−1.1 (2)
C12—C11—N2—O4	9.3 (2)	C7—C4—C5—C6	−177.24 (16)
C11—C10—C9—C14	2.1 (2)	C4—C3—C2—C1	0.4 (3)
C11—C10—C9—C8	178.34 (14)	C6—C1—C2—C3	−1.3 (3)
C10—C9—C14—C13	−2.3 (2)	N1—C1—C2—C3	−178.45 (16)
C8—C9—C14—C13	−178.65 (16)	C11—C12—C13—C14	0.6 (3)
C2—C1—N1—O1	−173.84 (17)	C9—C14—C13—C12	0.9 (3)
C6—C1—N1—O1	8.9 (2)	C10—C9—C8—C7	32.4 (3)
C2—C1—N1—O2	6.3 (2)	C14—C9—C8—C7	−151.46 (19)
C6—C1—N1—O2	−170.93 (15)	C9—C8—C7—C4	6.1 (3)
C10—C11—C12—C13	−0.8 (3)	C5—C4—C7—C8	−140.7 (2)
N2—C11—C12—C13	178.83 (14)	C3—C4—C7—C8	43.3 (3)
C5—C4—C3—C2	0.8 (3)		

### Hydrogen-bond geometry (Å, °)

**Table:**

*D*—H···*A*	*D*—H	H···*A*	*D*···*A*	*D*—H···*A*
C13—H13A···O1^i^	0.93	2.56	3.388 (2)	149

Symmetry codes: (i) *x*, *y*−1, *z*+1.
